# Supplementary material for: Using testing history to estimate HIV incidence in mothers living in resource-limited settings: Maximizing efficiency of a community health survey in Mozambique
Source: PLOS Glob Public Health. 2023 May 31;3(5):e0001628. doi: 10.1371/journal.pgph.0001628 (PMC10231780; doi:10.1371/journal.pgph.0001628)
Supplement: Attachment — Submitted filename: Response to Reviewers_sent.docx [file pgph.0001628.s002.docx]

Journal requirements

Journal Requirements:

1. Please review your reference list to ensure that it is complete and correct. If you have cited papers that have been retracted, please include the rationale for doing so in the manuscript text, or remove these references and replace them with relevant current references. Any changes to the reference list should be mentioned in the rebuttal letter that accompanies your revised manuscript. If you need to cite a retracted article, indicate the article’s retracted status in the References list and also include a citation and full reference for the retraction notice.

Response: The reference list has been reviewed. Not reference have been removed

2. Please amend your detailed Financial Disclosure statement. This is published with the article. It must therefore be completed in full sentences and contain the exact wording you wish to be published.

a. State the initials, alongside each funding source, of each author to receive each grant.
b. State what role the funders took in the study. If the funders had no role in your study, please state: “The funders had no role in study design, data collection and analysis, decision to publish, or preparation of the manuscript.”
c. If any authors received a salary from any of your funders, please state which authors and which funders.

If you did not receive any funding for this study, please simply state: “The authors received no specific funding for this work.”

Response: Please note that the funder statement is completed in full sentences and contain the exact wording specified by the funder institution. Please note that the grant was received by institutions and not by specific authors. We have specified the authors who received salary from the funders.

Additional Editor Comments (if provided):

Need some revision of the manuscript

Response: We have reviewed the manuscript in general and in particular for the specific comments. Thank you.

Response to Reviewers

**Reviewer #1:** The authors estimated the incidence of HIV among women who recently delivered in southern Mozambique using a cross-sectional household HIV-testing survey leveraging routine health card data on HIV testing to identify incident cases. Following are some minor comments to improve the clarity of the paper:

**1. Abstract:** “Most mothers (83.3%,1428/1714) had a documented HIV test result and date.” –Please clarify if the remaining 16.7% of the mothers had not been tested for HIV previously as HIV testing was offered to mothers with no prior HIV testing history too according to the Methods in the abstract.

Response: All the mothers included in this analysis of incidence (n=1714) were tested at the time of the survey (figure 1). Among the 1714 mothers included, 1594 (93.0%) showed a clinical document and in 1428 (83.3%) this document showed contained the date and result of the previous test. For the remaining 166 (16.7%) mothers with a previous negative test but without a registered date, we used the delivery as the last test date (methods, lines 80-82). The decision to use the delivery date as date of last test was in accordance with the national recommendation to test all HIV-negative mothers for HIV at delivery. We have rephrased the sentence in the abstract and we have added the national recommendation of testing at delivery in the methods section (line 80) for more clarification.

**2. Introduction,** lines 23-26: It is not clear if and how the current study used data from the rural Health Demographic Surveillance System. The abstract mentions a cross-sectional household HIV-testing survey.

Response: Indeed, the study uses data both from the HDSS and a cross sectional household HIV testing survey. We have rephrased the sentence in the introduction eliminating the reference to the HDSS maintaining the explanation of the HDSS in methods (lines 30-34).
We used HDSS data to select and locate 5000 households for the household survey, to ascertain the vital status when the mother was not located and also for verbal autopsy information among deceased mothers.

**3. Methods, Study design:**
- Please clarify if the “broader cross-sectional household survey” was the HDSS.

Response: This study used data from a cross-sectional household survey, which was conducted in the Manhiça District in October 2017- April 2018. In addition, we use data from the HDSS, which continuously documents vital events, including pregnancies, births, deaths, and migrations in the district. The cross-sectional study was performed as a different household visit, independent of the HDSS. We have rephrased the sentence in methods for clarification, in lines 45-46.

- Before applying a simple random sampling to identify 5000 children born alive, the first step is to identify and list all children born alive within the four years before study implementation. How many children in total were born alive during the four-year period?

Response: A total of children were born alive during the four-year period. We have added this to the study profile, according reviewer recommendation.

- Why were 5000 children identified, that is, how was the sample size calculated?

Response: This is indeed a study limitation highlighted (line 225), the sample size for HIV incidence estimation was not defined prior to the survey as the main objective was to estimate prevalence. For prevalence, sample size was calculated using a precision of 0.03 and 0.04 and assuming a prevalence of 10% and15%, for the children 0-23 months and 24-48 months of age, respectively

- Were there any household with more than one mother with a child born within the past four years? How many mothers per household were selected?

Response: These procedures are explained in more detail in the main manuscript referring to the cross-sectional study (reference number 12. Fuente-Soro L, et al. Community-based progress indicators for prevention of mother-to-child transmission and mortality rates in HIV). We have added the sentence: “for further information on broader cross-sectional household see Fuente-Soro et al. (12)” in line 53. To summarize: If two or more randomly selected children belong to the same household, or in the case of twins, only the first child randomly selected was included for participation. This was done in order to select independent mother-child pairs as opposed to having one mother who has multiple children within the desired age ranges participate in more than one mother-child pair.

- Was any HIV status determined by verbal autopsy alone in absence of documentation/health card? If yes, how reliable is that?

Response: For this sub-analysis of incidence, we only included mothers who were alive and tested at the survey (see lines 50-54)

- How many home visit attempts were made for the HIV test survey?

Response: For each participant, three household visit attempts were made before defining the status as absent. We have added this information in the methods section (lines 49-51).

.

**4. Methods,** **Study procedures:** Do the HIV rapid diagnostic tests from the two manufacturers have same diagnostic accuracy?

Response: The study procedures section (lines 62-66), highlights that we followed the national guidelines for HIV rapid diagnostic test in Mozambique. It consists on the Abbott Determine™ HIV 1/2 for initial testing and the Trinity Biotech Uni-Gold™ Recombigen® HIV test for confirmatory testing. This initial and confirmatory testing algorithm is used as point-of-care testing at health facilities within their laboratories and when appropriate by providers at service delivery points. In addition, this algorithm is used within voluntary counseling and testing centers, at the community level for community HIV testing campaigns, and to test health facility blood supplies.

**5. Methods,** **Statistical analysis:**
- For the second sensitivity analysis, women with a negative test within previous three months were excluded from the analysis. However, participants who “had tested HIV-negative more than three months before the study visit” were offered testing (lines 59-60) suggesting participants with a negative HIV test within last three months were not offered testing. Why? Please clarify.

Response: In the survey, participants with a negative HIV test within last three months were not offered testing, according to national guidelines, (methods section, lines 62-64).

- A sensitivity analysis excluding mothers without documented prior HIV test (prenatal card or child’s health card), and mothers without history of any prior HIV test or mothers without any antenatal visits during their last pregnancy will be useful.

Response: Thank you for your recommendation. According to peer reviewer suggestion, we have conducted a sensitivity analysis excluding the 120 mothers without documented prior HIV test was performed and HIV incidence (per 100 person-year) resulted 1.73 (95% CI: 1.27 – 2.35), which was not statically different to the HIV incidence reported in the manuscript. The sensitivity analysis result has been included in the limitations of the study.

**6. Results:**
− What proportion of the 2221 HIV-negative results were undocumented self-reported? How reliable is self-reporting? Is there any previous study on the validity of self-report of HIV testing?

Response: Thank you for your comment. Among the 1714 mothers included in the incidence analysis, 1594 (93.0%) showed documentation and 120(7.0%) reported a previous negative HIV test but did not show documentation (lines 119 -123, figure 1 and table 2). A total of 16 among the 120 (13.3%) who did not showed documentation resulted HIV positive at survey. Data from Manhiça in 2015 showed that more than one third of individuals testing HIV-positive did not disclosure their previous positive HIV diagnosis in the health facility (Fuente-Soro et al JIAS 2018). Therefore, we cannot guarantee new infection among this 16 of 1714 mothers (0.9%). However, after peer reviewer suggestion, we have conducted a sensitivity analysis excluding the 120 mothers without documented prior HIV test, and our result of HIV incidence did not significantly change. We have added this explanation in the limitations section (lines 232-239).

- The section ‘Clinical documentation showed at survey’ needs to be placed before the ‘Sociodemographic characteristics of mother-child pairs at survey’.

Response: We have placed “Clinical documentation showed at survey’ before the ‘Sociodemographic characteristics of mother-child pairs at survey’, as per reviewer recommendation.

**Reviewer #2**: I have made some of suggestions which wold help to upgrade paper quality. I have attached the file with this assignment. I have suggest some of correction between abstract and method section mainly. Some of analysis you may need to more clarify.

| **Variable** |  | **Text** | **Comment** |
| --- | --- | --- | --- |
| Abstract |  | Its over 250 words | Should be more concise |
| Background | Page 4 line 1 | Introduction | Change introduction to background |
| Methods | Page 5 | Inclusion and exclusion criteria | Clarify more specifically |
| Statistical interfaces |  |  | Clarify more specifically with test name |

**Response:**

Thank you for your comments. Please find bellow the answer to your comments:

**1)** The abstract has been reduced to less than 250 words.

**2)** Introduction has not been changed to background, following the editors recommendation in order to ensure journal requirements.

**3)** We have clarified in lines 55-60 the inclusion and exclusion criteria for incidence estimation. In summary, we included those mothers interviewed in the cross-sectional household study with a prior negative HIV test within the last four years and who received a new test at survey, and we excluded mothers without a previous negative HIV test or without a HIV test during the survey, as well as, caregivers other than mothers. (lines 55-60).

**4)** We have specified in lines 99-100 that the comparisons between groups were made using Pearson chi-square or Fisher exact test and Kruskal Wallis tests, as applicable, following your recommendation.
